# Supplementary figures and images for: Epiphytic diatom community structure and richness is determined by macroalgal host and location in the South Shetland Islands (Antarctica)
Source: PLoS One. 2021 Apr 30;16(4):e0250629. doi: 10.1371/journal.pone.0250629 (PMC8087030; doi:10.1371/journal.pone.0250629)

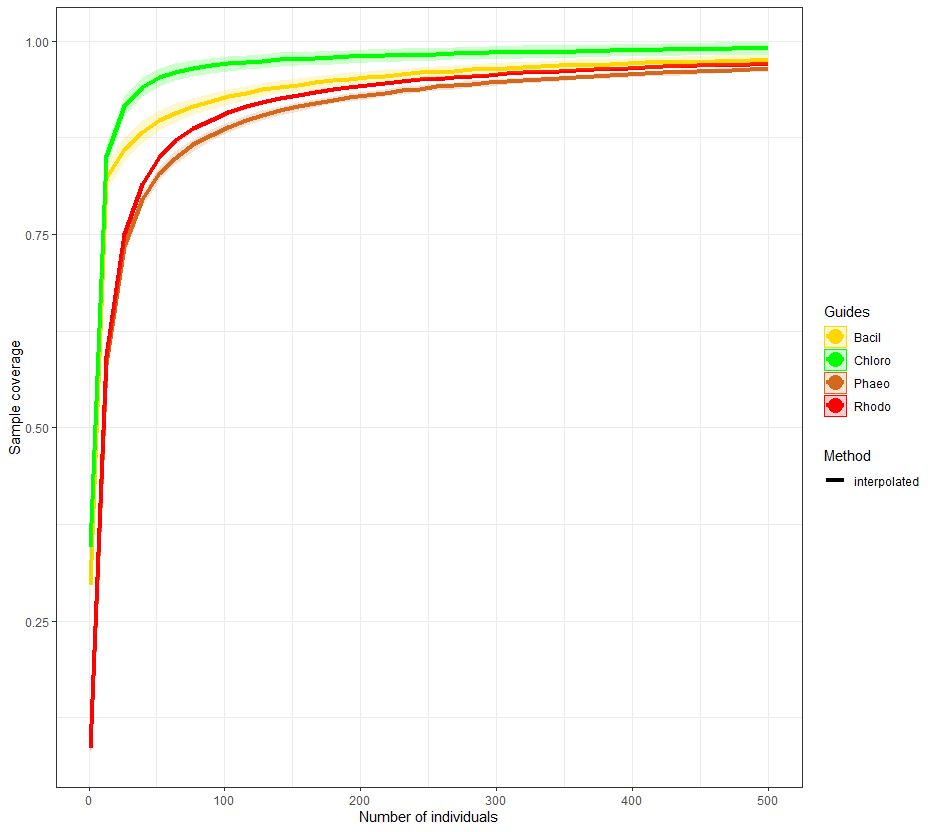

Supplement: S1 Fig — Rhodo = Rhodophyta, phaeo = Phaeophyceae, bacil = Bacillariophyceae, chloro = Chlorophyta. (TIF) [file pone.0250629.s001.tif]

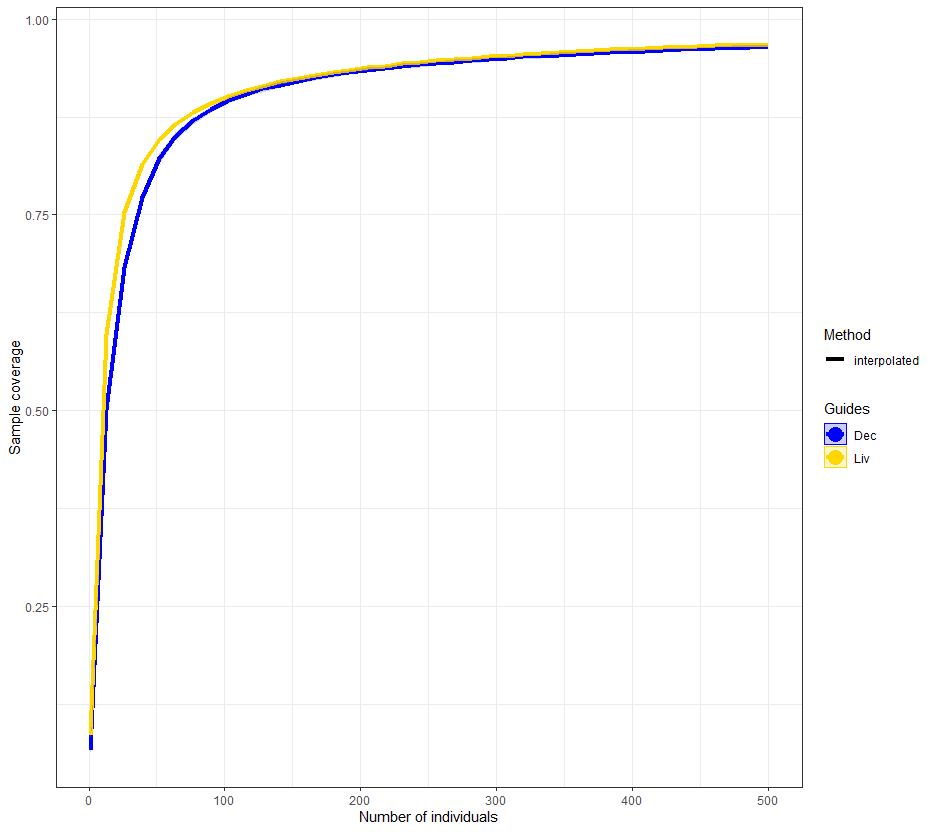

Supplement: S2 Fig — Dec = Deception island, Liv = Livingston island. (TIF) [file pone.0250629.s002.tif]
