# Supplementary material for: Epiphytic diatom community structure and richness is determined by macroalgal host and location in the South Shetland Islands (Antarctica)
Source: PLoS One. 2021 Apr 30;16(4):e0250629. doi: 10.1371/journal.pone.0250629 (PMC8087030; doi:10.1371/journal.pone.0250629)
Supplement: S1 Table — Pheaophyceae (Phaeo, n = 10), Rhodophyta (Rhodo, n = 25), Bacillariophyceae (Bac, n = 2) and Chlorophytas (Chlo, n = 1) have been investigated. Macroalgal host: Au = Adenocystis utricularis, Bc = Ballia callitricha, Cj = Cystosphaera jacquinotii, Dp = Delisea pulchra, Dan = Desmarestia anceps, Dant = Desmarestia antarctica, Ds = Desmarestia sp., Gs = Gigartina skottsbergii, Gt = Gymnogongrus turquettii, Hg = Himantothallus grandifolius, Ic = Iridaea cordata, Mh = Monostroma hariotii, Mm = Myriogramme cf. manginii, Pd = Palmaria decipiens, Pp = Piccionella plumosa, Pc = Plocamium cartilagineum, Ph = Plocamium cf. hookeri, Pe = Pyropia endiviifolia. Bold text shows first records in DI and LI. (DOCX) [file pone.0250629.s003.docx]

Supplement table S1 Epiphytic diatom composition and frequency on Antarctic macroalgae, specific proportion and occurrence on Deception (DI) and Livingston Island (LI). Pheaophytes (Phaeo, n=10), Rhodophytes (Rhodo, n=25), Bacillariophytes (Bac, n=2) and Chlorophytes (Chlo, n=1) have been investigated. Macroalgal host: Au = Adenocystis utricularis, Bc = Ballia callitriche, Cj = Cystosphaera jacquinotii, Dp = Delisea pulchra, Dan = Desmarestia anceps, Dant = Desmarestia Antarctica, Ds = Desmarestia sp., Gs = Gigartina skottsbergii, Gt = Gymnogongrus turquettii, Hg = Himantothallus grandifolius, Ic = Iridaea cordata, Mh = Monostroma hariotii, Mm = Myriogramme cf. manginii, Pd = Palmaria decipiens, Pp = Piccionella plumose, Pc = Plocamium cartilagineum, Ph = Plocamium cf. hookeri, Pe = Pyropia endiviifolia. Bold text shows first records in DI and LI.

| TAXA | % of total abundance | Host macroalga | Islands | Locations | % of samples | | | | |
| --- | --- | --- | --- | --- | --- | --- | --- | --- | --- |
|  |  |  |  |  | Phaeo | Rhodo | Bac | Chlo | All |
| *Achnanthes brevipes* C. Agardh | <1 - 7.8 | Pc, Dp, Da, Dan | DI LI | Base Moore / Polish / Raquelies | 40 | 8 | 0 | 0 | 15.8 |
| *Achnanthes vicentii* Manguin | <1 | Da | DI | Base | 10 | 0 | 0 | 0 | 2.6 |
| *Amphora cf. gourdonii* Peragallo | <1 – 1.6 | Hg, Pe, Ic, Mm, Bc, Pp, Dan, Bac, Gs, Da, Au | DI  LI | Base / Fildes / Fumaroles / Seals / Telephone/  Moore / Raquelies | 40 | 28 | 50 | 0 | 31.6 |
| *Amphora cf. racovitzae* Van Heurck | <1 | Au, Bc, Pp, Pd, Dan | DI  LI | Telephone  Moore / Raquelies | 20 | 12 | 0 | 0 | 13.2 |
| *Amphora Type C* (sensu Scott & Thomas 2005) | <1 | Pp | LI | Moore | 0 | 4 | 0 | 0 | 2.6 |
| *Berkeleya adeliensis* Medlin | 6.7 | Bac | DI | Fumaroles | 0 | 0 | 50 | 0 | 2.6 |
| *Berkeleya antarctica* Grunow | 3.7 | Bac | DI | Fumaroles | 0 | 0 | 50 | 0 | 2.6 |
| *Berkeleya rutilans* (Trentepohl Ex Roth) Grunow | 77.7 | Bac | DI | Fumaroles | 0 | 0 | 50 | 0 | 2.6 |
| *Brandinia cf. mosimaniae* Fernandes In Fernandes, Procopiak & Portinho | <1-70.5 | Au, Ic, Bac, Pd | DI  LI | Base / Fumaroles / Telephone  Raquelies | 10 | 8 | 100 | 0 | 13.2 |
| *Cf. Encyonema* | <1 | Gs | LI | Moore | 0 | 1 | 0 | 0 | 1 |
| *Campyloneis frenguelliae* Riaux-Gobin & J.M. Guerrero | <1 | Bc, Da, Pc | DI  LI | Fildes  Moore, Polish, Raquelies | 1 | 1 | 0 | 0 | 1.2 |
| *Cocconeis californica var. californica* Grunow In Van Heurck | <1 | Gt, Pc | LI | Moore / Polish | 0 | 8 | 0 | 0 | 5.3 |
| *Cocconeis antiqua* Tempere & Brun (Syn. *C. schuettii* Van Heurck) | <1 – 21.7 | Hg, Cj, Pc, Dp, Gt, Pe, Mm, Bc, Gs, Pd, Bac, Da, Dan, Ic | DI  LI | Base / Drum / Fumaroles / Seals / Telephone / Whaler’s  Raquelies / Moore / Polish | 50 | 64 | 50 | 0 | 57.9 |
| *Cocconeis californica* Grunow | <1 – 49.4 | Hg, Cj, Ds, Pc, Gs, Gt, Dp, Pe, Mm, Bc, Pp, Ph, Dan, Au | DI  LI | Whaler’s  Moore / Polish / Raquelies | 68 | 0 | 0 | 40 | 55.3 |
| *Cocconeis californica var. antarctica* Frenguelli & Orlando | <1 – 2.9 | Ic, Hg, Cj, Gs, Gt, Dp, Pe, Pc, Bc, Pp, Ph | DI  LI | Whaler’s  Moore / Polish | 10 | 44 | 0 | 0 | 31.6 |
| *Cocconeis californica var. kerguelensis* Heiden | <1 – 47.7 | Cj, Hg, Ic, Gs, Gt, Dp, Pc, Pe, Mh, Bc, Pp, Ph | DI  LI | Seal / Whaler’s  Moore / Polish | 20 | 64 | 0 | 0 | 50 |
| ***Cocconeis californica var. lengana* Rivera In Rivera, Parra & Gonzales** | **<1** | **Cj, Pe, Pp** | **DI**  **LI** | **Whaler’s**  **Moore** | **0** | **12** | **0** | **0** | **7.9** |
| *Cocconeis costata* Gregory | <1 – 24.5 | Ic, Hg, Dsp, Pc, Gk, Gt, Dp, Pc, Pe, Mh, Mm, Bc, Pp, Ph, Pd, Bac, Da, Dan, Au | DI  LI | Base / Drum / Fumaroles  Base / Moore / Polish / Raquelies | 90 | 92 | 50 | 100 | 89.5 |
| *Cocconeis costata var. antarctica* Manguin | <1 – 6.5 | Ic, Hg, Dsp, Pc, Gs, Gt, Dp, Pe, Mh, Mm, Bc, Pp, Ph, Pd, Da, Dant, Bac, Au | DI  LI | Seal  Moore / Polish | 0 | 36 | 0 | 0 | 23.7 |
| *Cocconeis costata var. hexagona* Grunow | <1 – 12.7 | Ic, Pc, Hg, Gs, Gt, Pe, Dp, Mm, Bc, Pp, Da, Dp, Ph, Dan, Pd | DI  LI | Fildes / Whaler’s  Base / Moore / Polish / Raquelies | 30 | 56 | 0 | 0 | 44.7 |
| ***Cocconeis curiosa* Hustedt** | **<1** | **Hg** | **DI** | **Whaler’s** | **10** | **0** | **0** | **0** | **2.6** |
| *Cocconeis dallmannii* Al-Handal, Riaux-Gobin, Romero & Wulff | <1 – 32.4 | Ic, Hg, Cj, Pc, Gs, Gt, Pe, Mh, Dp, Bc, Pp, Ph, Pd, Dan, Au | DI  LI | Seal / Telephone / Whaler’s  Base / Moore /Polish / Raquelies | 40 | 76 | 0 | 100 | 63.2 |
| ***Cocconeis distans* Gregory** | **<1 – 20.5** | **Hg, Cj, Dsp, Gt, Pc, Pe, Pc, Pp, Da, Dp, Ph, Dan,** | **DI**  **LI** | **Fildes / Whaler’s**  **Moore / Polish / Raquelies** | **50** | **40** | **0** | **100** | **39.5** |
| *Cocconeis fasciolata* (Ehrenberg) N. E. Brown *(syn. C. imperatrix* Schmidt*)* | <1 - 55 | Hg, Dsp, Pc, Gt, Dp, Pe, Mm, Bc, Ph, Pd, Dan, Bac, Da, Ic | DI  LI | Base / Drum / Fildes / /Fumaroles / Telephone / Whaler’s  Moore / Polish / Raquelies | 80 | 52 | 50 | 0 | 57.9 |
| ***Cocconeis melchiori* Frenguelli & Orlando** | **<1** | **Gs** | **LI** | **Polish** | **0** | **4** | **0** | **0** | **2.63** |
| *Cocconeis cf. melchioroides* Al-Handal, Riaux-Gobin, Romero & Wulff | <1 – 49.7 | Ic, Hg, Cj, Dsp, Pc, Gs, Gt, Dp, Pe, Mm, Bc, Pp, Da, Ph, Pd, Dan, Au | DI  LI | Fildes / Seal / Telephone / Whaler’s  Base / Moore / Polish / Raquelies | 60 | 92 | 0 | 0 | 76.3 |
| *Cocconeis neothumensis* Krammer | <1-1.2 | Gt, Pe, Mh, Da | DI  LI | Fildes  Moore | 10 | 8 | 0 | 100 | 10.5 |
| ***Cocconeis pinnata* Al-Handal, Riaux-Gobin, Romero & Wulff** | **<1** | **Hg, Gt, Dp, Ic, Pp, Gs, Pd, Au** | **DI**  **LI** | **Seal / Whaler’s**  **Base / Moore / Polish / Raquelies** | **20** | **32** | **0** | **100** | **26.3** |
| *Cocconeis pinnata var. Matsii* Al-Handal, Riaux-Gobin et Wulff | <1 – 26.1 | Ic, Hg, Pc, Gs, Gt, Dp, Pe, Mh, Mm, Bc, Pp, Da, Ph, Pd, Dan, Au | DI  LI | Base / Fildes / Seal / Telephone /Whaler’s  Base / Moore / Polish / Raquelies | 60 | 88 | 0 | 100 | 76.3 |
| ***Cocconeis pseudomarginata* Gregory** | **<1** | **Pp** | **LI** | **Moore** | **0** | **4** | **0** | **0** | **2.63** |
| ***Cocconeis reticulata var. deceptionis* Frenguelli & Orlando** | **<1 – 3.6** | **Pc, Gt, Dp, Mm, Da, Ph, Bac** | **LI** | **Polish** | **10** | **44** | **50** | **0** | **34.2** |
| *Cocconeis scutellum* Ehrenberg | <1 – 1.4 | Da, Gs, Gt, Pc, | DI  LI | Fildes  Moore / Polish | 10 | 88 | 0 | 0 | 13.2 |
| *Cocconeis scutellum f. stauroneiformis* Rabenhorst | <1 – 3.4 | Ic, Hg, Dsp, Gs, Dp, Pc, Mh, Pp, Da, Ph, Dan, Au | DI  LI | Base / Fildes / Whaler’s  Moore / Polish / Raquelies | 60 | 28 | 0 | 100 | 36.8 |
| *Cocconeis* sp. 1 | <1 – 5.5 | Ic, Pc, Dp, Bc, Pp, Dan | DI  LI | Fildes / Fumaroles  Moore / Polish / Raquelies | 10 | 56 | 0 | 0 | 39.5 |
| *Cocconeis* sp. 2 | <1 | Gt, Dp | DI  LI | Seal / Whaler’s  Base / Moore / Polish / Raquelies | 0 | 8 | 0 | 0 | 5.3 |
| *Coscinodiscus actinochilus* Ehrenberg | <1 | Pd, Bac | DI | Drum / Fumaroles | 0 | 4 | 50 | 0 | 5.3 |
| *Entopyla cf. ocellata* Ehrenberg | <1 | Gs | DI | Seal | 0 | 4 | 0 | 0 | 2.6 |
| *Eunotogramma cf. marginopunctata* Weisse | <1 | Gs | DI | Fumaroles | 0 | 4 | 0 | 0 | 2.6 |
| *Eupleuria ocellata* Arnott *(Syn. Entopyla ocellata* (Arnott) Grunow) | <1 | Dant, Pdec, | DI | Seal | 10 | 0 | 0 | 0 | 2.6 |
| *Fallacia marnieri* (Manguin) Witkowski, Lange-Bertalot & Metzelin | <1 | Au, Bac | DI  LI | Fumaroles / Whaler´s  Moore / Polish / Raquelies | 10 | 8 | 50 | 0 | 7.9 |
| *Fallacia* sp Sticke & Mann In Round, Crawford & Mann | <1 | Dant, Hg | LI | Moore / Polish / Raquelies | 30 | 24 | 50 | 0 | 26.3 |
| *Fragilaria* sp Lyngbye | <1 | Au, Pd, Bac | DI  LI | Drum / Fumaroles  Base / Raquelies | 10 | 8 | 50 | 0 | 10.5 |
| *Fragilaria striatula* Lyngbye | <1 | Hg, Bc, Gs | DI  LI | Fumaroles / Whaler’s  Moore | 10 | 8 | 50 | 0 | 10.5 |
| *Fragilariopsis curta* (Van Heurck) Hustedt | <1 | Au, Hg, Pd, Cj | DI  LI | Drum / Whaler’s  Raquelies | 20 | 8 | 0 | 0 | 10.5 |
| *Fragilariopsis kerguelensis (*O’Meara) Hustedt | <1 | Dp | LI | Polish | 0 | 4 | 0 | 0 | 2.6 |
| *Fragilariopsis nana* (Steemann Nielsen) Paasche | <1 | Dant, Dp | DI  LI | Drum  Raquelies | 10 | 4 | 0 | 0 | 5.3 |
| *Fragilariopsis rhombica* (O’Meara) Hustedt | <1 | Dant | LI | Raquelies | 10 | 0 | 0 | 0 | 2.6 |
| *Gomphonemopsis ligowskii* Al-Handall & E.W.Thomas | <1 – 61.6 | Hg, Danc, Dant, Au, Pc, Gt, Gs, Dp, Ph, Pd, Pe, Mm, Pp, Bc, | DI  LI | Base / Fildes / Seal / Telephone / Whaler’s  Base / Moore / Polish / Raquelies | 70 | 84 | 0 | 0 | 73.7 |
| *Gomphonemopsis* sp Medlin In Medlin & Round | <1 – 18.7 | Hg, Danc, Dp, Pc, Bc, Mm, Ph, Gs, Ic | DI  LI | Base / Fildes / Seal / Whaler’s  Moore / Polish | 30 | 32 | 0 | 100 | 31.6 |
| ***Gomphonemopsis pseudoexigua* (Simonsen) Medlin In Medlin & Round** | **<1** | **Dp, Pp** | **LI** | **Moore** | **0** | **8** | **0** | **0** | **5.2** |
| *Grammatophora angulosa* Ehrenberg | <1 – 5.7 | Au, Gs | DI  LI | Seal  Raquelies | 10 | 8 | 0 | 0 | 7.9 |
| *Grammatophora arctica* Cleve | <1 | Gs | DI | Seal | 0 | 4 | 0 | 0 | 2.6 |
| *Grammatophora arcuata* Ehrenberg | <1 | Gs | DI | Seal | 0 | 4 | 0 | 0 | 2.6 |
| *Grammatophora* Ehrenberg | <1 | Dant, Gs, Mm, Pc | DI  LI | Seal  Moore / Raquelies | 10 | 12 | 0 | 0 | 10.5 |
| *Gyrosigma* sp Hassall | <1 | Pp | LI | Moore | 0 | 4 | 0 | 0 | 2.6 |
| *Halamphora* sp (Cleve) Levkov | <1 | Hg, Dant, Danc, Ic, gt | DI  LI | Base / Fildes / Whaler’s  Polish / Raquelies | 30 | 8 | 0 | 0 | 13.2 |
| *Halamphora acutiuscula* (Kützing) Levkov | <1 – 1.3 | Dant, Gs, Ic | DI  LI | Seal  Moore / Raquelies | 10 | 8 | 0 | 0 | 7.9 |
| *Licmophora* sp Agardh | <1 | Dsp, Au, Dant, Danc, Pp, Ic, Pe | DI  LI | Fildes / Whaler’s  Moore / Raquelies | 40 | 12 | 0 | 0 | 18.4 |
| *Licmophora antarctica* Peragallo | <1 – 1.8 | Au, Bc, Danc, Dant, Dp, Gt, Gs, Hg, Pd, Pe | DI  LI | Base / Fildes  Base / Moore / Polish/ Raquelies | 50 | 28 | 0 | 0 | 31.6 |
| *Licmophora gracilis* (Ehrenberg) Grunow | <1 – 54.4 | Danc, Dant, Gs, Pd | DI  LI | Base / Seal  Base / Moore / Raquelies | 40 | 8 | 50 | 100 | 21.1 |
| *Licmophora luxuriosa* Heiden & Kolbe | <1 | Pd | LI | Base / Moore | 0 | 4 | 0 | 100 | 5.3 |
| *Luticola* sp Mann in Round, Crawford & Mann | <1 | Au | LI | Raquelies | 10 | 0 | 0 | 0 | 2.6 |
| *Lyrella* sp Karayeva | <1 | Bac | DI | Fumaroles | 0 | 0 | 50 | 0 | 2.6 |
| *Melosira adeliae* Manguin | <1 – 16.6 | Danc, Dant, Hg, Pd, Bac | DI | Base / Drum / Fumaroles / Telephone / Whaler’s | 30 | 8 | 100 | 0 | 18.4 |
| *Navicula* sp Bory de Saint-Vincent | <1 – 2.6 | Au, Danc, Dant, Dsp, Hg, Ic, Cj, Gt, Gs, Pd, Dp, Pc, Mm, Bc, Bac | DI  LI | Base / Drum / Fumaroles / Seal / Telephone / Whaler’s  Moore / Polish / Raquelies | 60 | 52 | 100 | 0 | 55.3 |
| *Navicula criophila* (Castracane) De Toni | <1 – 2.3 | Au, Dant, Pd, Dp, Pp, Bc, Pc, Gt, Pe | DI  LI | Telephone  Base / Moore / Polish / Raquelies | 20 | 40 | 0 | 0 | 31.6 |
| ***Navicula cristata* Ehrenberg** | **<1 – 2.2** | **Danc,Dant, Hg, Ic, Cj, Pc, Gt, Dp, Bc, Pp, Gs, Ph, Pd** | **DI**  **LI** | **Base/ Fildes / Seal / Whaler’s**  **Base / Moore / Polish / Raquelies** | **40** | **56** | **0** | **0** | **47.4** |
| *Navicula directa* (W.Smith) Ralfs | <1 – 2.9 | Au, Danc, Dant, Hg, Pd, Gs, Pp, Bc, Mm Ic, Pc, Dp, Pe, Gt | DI  LI | Base / Drum / Fumaroles / Seal / Telephone  Base / Moore / Polish / Raquelies | 40 | 56 | 0 | 0 | 50 |
| ***Navicula delognei* Van Heurck** | **<1** | **Au, Dant, Dp** | **LI** | **Polish / Raquelies** | **20** | **4** | **0** | **0** | **7.9** |
| *Navicula gelida* Grunow | <1 – 1.4 | Au, Dant, Hg, Ic, Pd, Gs, Gt, Pc, Pe, Mm, Bc, Pp, Bac | DI  LI | Base / Drum / Seal / Telephone / Whaler’s  Base / Moore / Polish / Raquelies | 30 | 52 | 50 | 0 | 76.3 |
| *Navicula glaciei* Van Heurck | <1 – 31.4 | Au, Dant, Anc, Hg, Dsp, Bac, Ic, Pd, Ph, Dp, Gs, Pc, Gt, Pe, Pc, Pp, Bc, Mm | DI  LI | Base / Drum / Fildes / Fumaroles / Telephone / Whaler’s  Base / Moore / Polish / Raquelies | 90 | 72 | 100 | 0 | 44.7 |
| ***Navicula gracilis* Ehrenberg** | **<1** | **Dant, Dsp, Bc, Gt, Dp** | **DI**  **LI** | **Fumaroles / Whaler’s**  **Moore / Polish / Raquelies** | **20** | **12** | **50** | **0** | **15.8** |
| *Navicula incertata* Lange-Bertalot | <1 – 21.7 | Au, Dant, Anc, Hg, Dsp, Bac, Ic, Pd, Ph, Dp, Gs, Pc, Gt, Pe, Pc, Pp, Bc, Mm, Mh, Bac | DI  LI | Base / Drum / Fildes / Fumaroles /Seal / Telephone / Whaler’s  Base / Moore / Polish / Raquelies | 100 | 92 | 100 | 100 | 94.7 |
| *Navicula jejunoides* van Heurck | <1 | Hg, Gs, Mm, Pd, Ic, Pc, Gt, Ic | DI  LI | Drum / Seal / Whaler’s  Moore / Polish | 10 | 32 | 0 | 0 | 23.7 |
| *Navicula perminuta* Grunow | <1 – 64.2 | Au, Dant, Anc, Hg, Dsp, Bac, Ic, Pd, Ph, Dp, Gs, Pc, Gt, Pe, Pc, Pp, Bc, Mm, Mh, Bac | DI  LI | Base / Drum / Fildes / Fumaroles / Seal / Telephone /Whaler’s  Base / Moore / Polish / Raquelies | 100 | 100 | 100 | 100 | 100 |
| ***Navicula schefterae* Lobban** | **<1 – 2.9** | **Dant, Danc, Dsp, Hg, Pd, Gs, Ic, Pp, Bc, Mm, Dp, Gt** | **DI**  **LI** | **Base / Fildes / Seal / Telephone / Whaler’s**  **Moore / Polish / Raquelies** | **40** | **36** | **0** | **0** | **32.2** |
| *Naviculaceae* Kützing | <1 – 2.7 | Au, Danc, Dant, Hg, Dp, Bc, Mm, | DI  LI | Base  Moore / Polish / Raquelies | 40 | 16 | 0 | 0 | 21.1 |
| *Neomoelleria Antarctica* (Castrac.) S.Blanco & C.E.Wetzel *(syn. Eucampia antarctica (Castrac.) Mangin)* | <1 | Dant, Gs, Pd | DI  LI | Drum / Telephone  Raquelies | 10 | 8 | 0 | 0 | 7.9 |
| ***Nitzschia bicapitata* Cleve** | **<1** | **Bac** | **DI** | **Fumaroles** | **0** | **0** | **50** | **0** | **2.6** |
| *Nitzschia* sp Hassall | <1 – 1.1 | Au, Dant, Cj, Gt, Mm | DI  LI | Fumaroles / Whaler’s  Moore / Raquelies | 20 | 12 | 50 | 0 | 15.8 |
| ***Nitzschia decipiens* Hustedt** | **<1** | **Au** | **LI** | **Raquelies** | **10** | **0** | **0** | **0** | **2.6** |
| *Nitzschia hybrida* Grunow | <1 – 1.2 | Au, Dant, Cj, Bc | DI  LI | Fumaroles / Whaler’s  Moore / Raquelies | 20 | 8 | 50 | 0 | 13.6 |
| *Nitzschia lecointei* van Heurck | <1 – 2.5 | Au, Dant, Ic | LI | Moore / Raquelies | 20 | 4 | 0 | 0 | 7.9 |
| *Nitzschia stellata* Manguin | <1 | Bac | DI | Base | 0 | 0 | 100 | 0 | 2.6 |
| *Nitzschia taenia* Hustedt | <1 | Pp | LI | Moore | 0 | 4 | 0 | 0 | 2.6 |
| *Odontella litigiosa* (Van Heutck) Hoban | <1 | Pd, Bac | DI | Drum / Fumaroles / Telephones | 0 | 8 | 100 | 0 | 7.9 |
| *Parlibellus cruciculus* (Smith) Witkowski, Lange-Bertalot & Metzelin | <1 – 2.9 | Danc, Bac | DI | Base / Fumaroles | 10 | 0 | 100 | 0 | 5.3 |
| *Pinnularia* sp Ehrenberg | <1 | Au | LI | Raquelies | 10 | 0 | 0 | 0 | 2.6 |
| *Pinnularia quadratarea* (Schmidt) Cleve | <1 | Pe | LI | Moore | 0 | 4 | 0 | 0 | 2.6 |
| *Planothidium delicatulum* Round & Bukhtiyarova | <1 – 1.2 | Dant, Ic, Hg, Pd, Pp, Cj, Bac | DI  LI | Base / Fumaroles / Telephone / Whaler’s  Moore / Raquelies | 20 | 16 | 100 | 0 | 18.4 |
| *Planothidium dubium* (Grunow) Round & Bukhtiyarova | <1 | Au, Ic, Pd, Pp, Bc, Bac | DI  LI | Drum / Fumaroles / Whaler’s  Moore / Raquelies | 10 | 12 | 100 | 0 | 13.2 |
| *Planothidium sp* Round & Bukhtiyarova | <1 – 1.1 | Bc, Danc, Dant, Dp, Mh, Pc, Pd, Pp | DI  LI | Base / Drum / Telephone  Moore / Polish / Raquelies | 30 | 28 | 0 | 100 | 28.9 |
| *Porosira cf pseudodenticulata* Jorgensen | <1 – 3.5 | Au, Pc | LI | Moore / Raquelies | 10 | 4 | 0 | 0 | 5.3 |
| ***Pseudogomphonema arcticum* (Grunow) Medlin** | **<1 – 5.9** | **Danc, Dant, Cj, Gt, Pe, Pc, Ic, Mm, Gs, Pd** | **DI**  **LI** | **Base / Drum / Seal / Telephone / Whaler’s**  **Moore** | **20** | **48** | **0** | **0** | **36.8** |
| ***Pseudogomphonema groenlandicum* (Ostrup) Medlin** | **<1 – 1.7** | **Danc, Dant, Dsp, Hg, Dp, Pe, Pc, Ic, Mm, Gs, Pd, Bac, Mh** | **DI**  **LI** | **Base / Drum / Fildes/ Seal / Telephone / Whaler’s**  **Base / Moore /Polish** | **60** | **44** | **50** | **100** | **50** |
| *Pseudogomphonema kamtschaticum* (Grunow) Medlin | <1 | Au, Dant, Danc, Hg, Dsp, Bac, Ic, Pd, Ph, Dp, Gs, Pc, Gt, Pe, Pc, Pp, Bc, Mm, Mh, Bac | DI  LI | Base / Drum / Fildes / Fumaroles / Seal / Telephone / Whaler’s  Base / Moore / Polish / Raquelies | 100 | 96 | 100 | 100 | 97.3 |
| *Pseudogomphonema sp* Medlin in Medlin & Round | <1 – 28.2 | Danc, Dant, Dsp, Hg, Cj, Gt, Pe, Dp, Pc, Ic, Mm, Mh | DI  LI | Base / Fildes / Whaler’s  Moore / Polish / Raquelies | 40 | 32 | 0 | 100 | 34.2 |
| ***Pseudogomphonema* sp. 1** | **<1 – 59.1** | **Au, Danc, Dant, Dsp, Hg, Ic, Pd, Gs, Ph, Dp, Cj, Pc, Gt, Pe, Bc, Pp, Bac** | **DI**  **LI** | **Base / Fildes / Fumaroles / Seal / Telephone / Whaler’s**  **Base / Moore / Polish / Raquelies** | **90** | **76** | **50** | **0** | **76.3** |
| ***Pseudogomphonema* sp. 2** | **<1** | **Danc, Ic** | **DI** | **Base** | **10** | **4** | **0** | **0** | **5.3** |
| *Pseudostaurosira brevistriata* (Grunow) Williams & Round | <1 | Au, Dant, Hg, Ic, Pd, Pe, Bac | DI  LI | Base / Fildes / Fumaroles / Telephone / Whaler’s  Base / Moore / Raquelies | 40 | 16 | 100 | 0 | 26.3 |
| *Pseudostaurosira brevistriata var. brevistriata* | <1 | Pd | DI | Drum | 0 | 4 | 0 | 0 | 2.6 |
| *Pseudostaurosira* sp Williams & Round | <1 | Au, Danc, Dant | DI  LI | Base  Raquelies | 30 | 0 | 0 | 0 | 7.9 |
| *Rhabdonema* sp*.* Kützing | <1 | Pd | DI | Drum | 0 | 4 | 0 | 0 | 2.6 |
| *Rhabdonema arcuatum* (Lyngbye) Kützing | <1 – 3 | Pd | DI | Drum | 0 | 4 | 0 | 0 | 2.6 |
| *Rhoicosphenia* sp Grunow | <1 – 2.6 | Danc, Hg | DI  LI | Base / Seal / Whaler’s  Base / Moore / Polish / Raquelies | 30 | 44 | 0 | 0 | 39.5 |
| *Rhoicosphenia abbreviata* (Agardh) Lange-Bertalot | <1 | Au, Danc, Dant, Hg, Gs, Gt, Pp | DI  LI | Fildes / Seal / Whaler’s  Moore / Polish / Raquelies | 40 | 16 | 0 | 100 | 21.1 |
| *Rhoicosphenia genuflexa* (Kützing) Medlin | <1 – 22.7 | Au, Dant, Hg, Pc, Gt, Dp, Mm, Bc, Pp, Ph, Gs | DI  LI | Seal / Whaler’s  Moore / Polish / Raquelies | 40 | 52 | 0 | 0 | 44.7 |
| *Rhoicosphenia linearis* Ostrup | <1 | Hg, Gt, Dp, Pc, Pe, Bc, Gs | DI  LI | Drum / Seal / Whaler’s  Moore / Polish | 10 | 40 | 0 | 0 | 28.9 |
| *Rhoicosphenia* sp. 1 | <1 | Dp, Pc, Pe | LI | Moore / Polish | 0 | 12 | 0 | 0 | 7.9 |
| *Sellaphora* sp Mereschkowsky | <1 | Au | LI | Raquelies | 10 | 0 | 0 | 0 | 2.6 |
| *Stauroneis* sp Ehrenberg | <1 - 2.9 | Bac | DI | Fumaroles | 0 | 0 | 50 | 0 | 2.6 |
| *Synedropsis* sp Hasle, Medlin & Syvertsen | <1 | Dant | DI  LI | Base  Raquelies | 10 | 0 | 50 | 0 | 5.3 |
| *Synedropsis fragilis* Hasle, Medlin & Syvertsen | <1 | Pe, Bac | DI | Moore | 0 | 4 | 50 | 0 | 2.6 |
| *Synedropsis recta* Hasle, Medlin & Syvertsen | <1 | Danc, Dant, Hg, Pe, Pc, Ic, Dp, Pd | DI, LI | Base / Drum  Moore / Polish / Raquelies | 40 | 20 | 0 | 0 | 23.7 |
| *Tabularia* sp (Kützing) Williams & Round | <1 – 2.2 | Hg | DI | Fildes | 10 | 0 | 0 | 0 | 2.6 |
| *Tabularia tabulata* (Agardh) Snoeijs | <1 - 74.3 | Au, Danc, Hg, Gs, Pd, Pp, Bc, Mm, Pc, | DI  LI | Base / Fildes / Seal / Telephone  Moore / Raquelies | 30 | 24 | 0 | 0 | 23.7 |
| ***Thalassionema nitzschioides* (Grunow) Mereschkowsky** | **<1** | **Dant** | **LI** | **Raquelies** | **10** | **0** | **0** | **0** | **2.6** |
| *Thalassiosira* sp Cleve | <1 | Hg, Cj, Pd, Pp | DI  LI | Whaler’s  Base / Moore | 10 | 12 | 0 | 0 | 10.5 |
| *Thalassiosira lentiginosa* (Janisch) Fryxell | <1 | Bac | DI | Fumaroles | 0 | 0 | 50 | 0 | 2.6 |
| *Thalassiosira oestrupii* (Ostenfeld) Hasle | <1 | Au | LI | Raquelies | 10 | 0 | 0 | 0 | 2.6 |
| *Trachyneis aspera* (Ehrenberg) Hasle | <1 | Danc, Gs, Bc | DI  LI | Fildes / Seal  Moore | 10 | 8 | 0 | 0 | 7.9 |
| *Trigonium arcticum* (Brightwell) Cleve | <1 | Gs | DI | Seal | 0 | 4 | 0 | 0 | 2.6 |
| *Total number of taxa* | 130 |  |  |  | 92 | 107 | 42 | 19 |  |
