# Supplementary material for: Epiphytic diatom community structure and richness is determined by macroalgal host and location in the South Shetland Islands (Antarctica)
Source: PLoS One. 2021 Apr 30;16(4):e0250629. doi: 10.1371/journal.pone.0250629 (PMC8087030; doi:10.1371/journal.pone.0250629)
Supplement: S2 Table — (DOCX) [file pone.0250629.s004.docx]

S 2 SIMPER analysis of comparison of predominant diatom species in LI and DI

| Species | Av. Abundances | | Av. diss | Diss/ SD | Contrib % | Cum % |
| --- | --- | --- | --- | --- | --- | --- |
|  | **LI** | **DI** |  |  |  |  |
| *Navicula perminuta* | 14.98 | 17.04 | 8.02 | 1.01 | 11.34 | 11.34 |
| *Gomphonemopsis ligowskii* | 10.98 | 6.16 | 6.95 | 0.99 | 9.83 | 21.17 |
| *Pseudogomphonema* sp. 1 | 12.56 | 8.16 | 6.39 | 1.00 | 9.03 | 30.20 |
| *Cocconeis melchioroides* | 13.13 | 2.76 | 6.36 | 0.87 | 9.00 | 39.21 |
| *Other* | 10.37 | 11.76 | 4.00 | 1.25 | 5.66 | 44.86 |
| *Cocconeis fasciolata* | 0.97 | 7.65 | 3.91 | 0.56 | 5.52 | 50.39 |
| *Cocconeis californica var. kerguelensis* | 7.40 | 0.83 | 3.79 | 0.57 | 5.35 | 55.74 |
| *Pseudogomphonema kamtschaticum* | 5.57 | 6.22 | 3.41 | 1.00 | 4.82 | 60.56 |
| *Cocconeis californica* | 2.49 | 3.67 | 2.79 | 0.47 | 3.95 | 64.51 |
| *Cocconeis dallmannii* | 5.24 | 0.99 | 2.79 | 0.64 | 3.94 | 68.45 |
| *Berkeleya rutilans* | 0.00 | 5.18 | 2.59 | 0.27 | 3.66 | 72.12 |
