# Supplementary material for: Epiphytic diatom community structure and richness is determined by macroalgal host and location in the South Shetland Islands (Antarctica)
Source: PLoS One. 2021 Apr 30;16(4):e0250629. doi: 10.1371/journal.pone.0250629 (PMC8087030; doi:10.1371/journal.pone.0250629)
Supplement: S3 Table — (DOCX) [file pone.0250629.s005.docx]

Supplement table S3 SIMPER analysis of comparison of epiphytic diatom communities in the South Shetland Islands (SSI, n=4) and Vestfold Hills (VH, n=1)

|  | Group SSI | Group VH |  |  |  |  |
| --- | --- | --- | --- | --- | --- | --- |
|  | Av. abundance | Av. abundance | Av. Diss | Diss/ SD | Contrib % | Cum % |
| *Navicula perminuta* | 52.75 | 0 | 12.55 | 1.32 | 12.69 | 12.69 |
| *Cocconeis melchioroides* | 51.25 | 0 | 11.83 | 1.94 | 11.97 | 24.65 |
| *Cocconeis dalmannii* | 27.75 | 0 | 6.07 | 1.15 | 6.14 | 30.80 |
| *Pseudogomphonema* sp. 1 | 22.50 | 0 | 5.29 | 0.77 | 5.35 | 36.15 |
| *Cocconeis calfornica var. kerguelensis* | 16.75 | 0 | 4.45 | 0.66 | 4.50 | 40.65 |
| *Licmophora kamtschaticum* | 16.75 | 0 | 3.92 | 0.92 | 3.97 | 44.61 |
| *Cocconeis fasciolata* | 16.75 | 0 | 3.41 | 0.87 | 3.45 | 48.06 |
| *Cocconeis californica* | 16.50 | 0 | 3.05 | 0.92 | 3.09 | 51.15 |
| *Grammatophora arctica* | 11.75 | 0 | 2.83 | 0.52 | 2.86 | 57.05 |
| *Brandinia* | 18 | 0 | 2.80 | 0.54 | 2.83 | 59.88 |
| *Tabularia tabulata* | 18 | 0 | 2.79 | 0.53 | 2.82 | 62.70 |
| *Fragilariopsis sublinearis* | 11.25 | 0 | 2.74 | 0.50 | 2.77 | 65.47 |
| *Berkeleya rutilans* | 17.50 | 0 | 2.66 | 0.5 | 2.69 | 68.16 |
| *Cocconeis antiqua* | 12 | 0 | 2.25 | 0.56 | 2.28 | 70.44 |
