# Supplementary material for: Epiphytic diatom community structure and richness is determined by macroalgal host and location in the South Shetland Islands (Antarctica)
Source: PLoS One. 2021 Apr 30;16(4):e0250629. doi: 10.1371/journal.pone.0250629 (PMC8087030; doi:10.1371/journal.pone.0250629)
Supplement: S4 Table — (DOCX) [file pone.0250629.s006.docx]

Supplement table S4 SIMPER analysis of comparison of diatom communities in the South Shetland Islands (SSI, n= 4) and MacMurdo Sound (MMS, n = 4).

|  | Group SSI | Group MMS |  |  |  |  |
| --- | --- | --- | --- | --- | --- | --- |
|  | Av. abundance | Av. abundance | Av. Diss | Diss/ SD | Contrib % | Cum % |
| *Fragilaripsis nana* | 0.5 | 72.50 | 4.50 | 3.85 | 5.59 | 5.59 |
| *Cocconeis fasciolata* | 16.75 | 78.75 | 3.92 | 2.19 | 4.87 | 10.45 |
| *Cocconeis melchioroides* | 51.25 | 3.50 | 3.51 | 1.58 | 4.36 | 14.82 |
| *Synedropsis recta* | 1 | 56.75 | 3.16 | 1.85 | 3.93 | 18.74 |
| *Navicula jejunoides* | 0.75 | 51.05 | 3.07 | 3.42 | 3.81 | 22.55 |
| *Melosira adeliae* | 0.50 | 53.90 | 2.96 | 1.54 | 3.67 | 26.22 |
| *Pseudogomphonema kamtschaticum* | 16.75 | 64.10 | 2.92 | 1.70 | 3.63 | 29.85 |
| *Navicula glaciei* | 0.50 | 45.33 | 2.90 | 1.58 | 3.60 | 33.45 |
| *Achnanthes vicentii* | 0.75 | 44.80 | 2.59 | 2.38 | 3.21 | 36.66 |
| *Cocconeis antiqua* | 12 | 36.90 | 2.03 | 1.52 | 2.52 | 42.19 |
| *Cocconeis dallmanni* | 27.75 | 0 | 1.95 | 1 | 2.42 | 44.61 |
| *Navicula perminuta* | 52.75 | 85 | 1.89 | 0.85 | 2.35 | 46.96 |
| *Tabularia tabulata* | 18 | 24.55 | 1.81 | 1.23 | 2.24 | 49.20 |
| *Pseudogomphonema* sp. 1 | 22.50 | 0 | 1.59 | 0.85 | 1.98 | 51.18 |
| *Nitzschia lecointei* | 0.50 | 24.80 | 1.48 | 1.57 | 1.84 | 53.02 |
| *Cocconeis californica* | 16.50 | 18.43 | 1.48 | 0.90 | 1.84 | 54.86 |
| *Licmophora gracilis* | 23 | 0.80 | 1.44 | 0.91 | 1.79 | 56.65 |
| *Parlibellus cruciculus* | 0.50 | 25.30 | 1.40 | 0.93 | 1.74 | 58.39 |
| *Fragilariopsis curta* | 0.75 | 29.70 | 1.31 | 0.85 | 1.62 | 60.01 |
| *Cocconeis californica var. kerguelensis* | 16.75 | 0.55 | 1.24 | 0.74 | 1.53 | 61.54 |
| *Brandinia* | 18 | 4.30 | 1.15 | 0.60 | 1.43 | 62.97 |
| *Fragilariopsis sublinearis* | 11.25 | 11.10 | 1.10 | 0.79 | 1.37 | 64.34 |
| *Berkeleya rutilans* | 17.50 | 0 | 1.07 | 0.53 | 1.33 | 65.67 |
| *Nitzschia medioconstricta* | 0.25 | 21.55 | 1.02 | 0.95 | 1.26 | 66.93 |
| *Navicula directa* | 12 | 10.30 | 0.97 | 0.80 | 1.20 | 68.14 |
| *Fragilaria islandica var. adeliae* | 0.25 | 16.10 | 0.93 | 1.60 | 1.15 | 69.29 |
| *Cocconeis deceptionensis* | 0.50 | 19.38 | 0.92 | 0.98 | 1.15 | 70.44 |
