# Supplementary material for: Epiphytic diatom community structure and richness is determined by macroalgal host and location in the South Shetland Islands (Antarctica)
Source: PLoS One. 2021 Apr 30;16(4):e0250629. doi: 10.1371/journal.pone.0250629 (PMC8087030; doi:10.1371/journal.pone.0250629)
Supplement: S5 Table — (DOCX) [file pone.0250629.s007.docx]

Supplement table S5 SIMPER analysis of comparison of epiphytic diatom communities in the Vestfold Hills (VH, n=1) and MacMurdo Sound (MMS, n=4)

|  | Group VH | Group MMS |  |  |  |  |
| --- | --- | --- | --- | --- | --- | --- |
|  | Av. abundance | Av. abundance | Av. Diss | Diss/ SD | Contrib % | Cum % |
| *Navicula perminuta* | 0 | 85 | 8.90 | 2.24 | 8.95 | 8.95 |
| *Cocconeis fasciolata* | 0 | 78.75 | 7.63 | 3.71 | 7.67 | 16.62 |
| *Fragilariopsis nana* | 0 | 72.50 | 6.90 | 3.02 | 6.93 | 23.55 |
| *Pseudogomphonema kamtschaticum* | 0 | 64.10 | 5.49 | 2.94 | 5.51 | 29.06 |
| *Synedropsis recta* | 0 | 56.75 | 4.68 | 1.97 | 4.70 | 33.76 |
| *Achnanthes vicentii* | 0 | 45.33 | 4.66 | 1.24 | 4.68 | 38.44 |
| *Navicula jejunoides* | 0 | 51.05 | 4.63 | 3.76 | 4.65 | 43.09 |
| *Melosira adeliae* | 0 | 53.90 | 4.16 | 1.40 | 4.18 | 47.28 |
| *Navicula glaciei* | 0 | 44.80 | 3.89 | 2.53 | 3.91 | 51.19 |
| *Achnanthes brevipes* | 0 | 49.40 | 3.29 | 0.83 | 3.31 | 54.50 |
| *Cocconeis antiqua* | 0 | 36.90 | 3.29 | 1.44 | 3.31 | 57.80 |
| *Cocconeis californica* | 0 | 18.43 | 2.98 | 0.70 | 2.99 | 60.79 |
| *Nitzschia lecointei* | 1 | 24.80 | 2.22 | 1.29 | 2.23 | 63.02 |
| *Parlibellus cruciculus* | 0 | 25.30 | 1.97 | 0.78 | 1.98 | 65.00 |
| *Cocconeis scutellum f. stauroneiformis* | 0 | 16.53 | 1.91 | 1.10 | 1.91 | 66.92 |
| *Tabularia tabulata* | 0 | 24.55 | 1.87 | 1.34 | 1.88 | 68.80 |
| *Fragilariopsis curta* | 0 | 29.70 | 1.76 | 0.84 | 1.77 | 70.57 |
