# Supplementary material for: Epiphytic diatom community structure and richness is determined by macroalgal host and location in the South Shetland Islands (Antarctica)
Source: PLoS One. 2021 Apr 30;16(4):e0250629. doi: 10.1371/journal.pone.0250629 (PMC8087030; doi:10.1371/journal.pone.0250629)
Supplement: S6 Table — * = contains only macroalgal information, not Bacillariophyceae. | = total data of the study. (DOCX) [file pone.0250629.s008.docx]

Supplement table S6 Diversity/Entropy indices of the compared studies. * = contains only macroalgal information, not bacillariophytes. | = total data of the study.

| Position in Antarctica | Location | Species richness (S) | Shannon diversity (H’) | N | Number of hosts species (genera) | Number of diatom species (genera) | Study |
| --- | --- | --- | --- | --- | --- | --- | --- |
| South Shetland Islands | **Deception Island** | 94 | 3.16 | 15 | 8* (6*) \| 18 | 94 (37) \| 129 (44) | This study |
|  | **Livingston island** | 88 | 2.90 | 23 | 15 (14) \| 18 | 82 (31) \| 129 (44) | This study |
|  | **King George island (Potter Cove)** | 47 | 2.63 | 19 | 19 (16) | 50 (29) | Al Handall & Wulff 2008 |
|  | **King George island (Admiralty bay) 2015** | 45 | 2.64 | 12 | 1 (1) | 46 \| 72 (32) | Majewska et al 2015 |
| McMurdo Sound | **Terra Nova Bay 2013** | 51 | 2.93 | 37 | 3 (3) | 73 (32) | Majewska et al 2013a |
|  | **Terra Nova Bay 2015** | 68 | 3.34 | 15 | 1 (1) | 57 \| 72 (32) | Majewska et al 2015 |
|  | **Terra Nova Bay 2014** | 36 | 3.04 | 10 | 1 (1) | 72 (30) | Majewska et al 2014 |
|  | **TNB + Cape Evans 2016** | 84 | 3.87 | 46 | 3 (3) | 109 (44) | Majewska et al 2016 |
| Vestfold Hills | **Davis station** | 6 | 1.79 | 15 | 16 (16) | 9 (9) | Thomas & Jiang 1986 |
