# Supplementary material for: Epiphytic diatom community structure and richness is determined by macroalgal host and location in the South Shetland Islands (Antarctica)
Source: PLoS One. 2021 Apr 30;16(4):e0250629. doi: 10.1371/journal.pone.0250629 (PMC8087030; doi:10.1371/journal.pone.0250629)
Supplement: S7 Table — (DOCX) [file pone.0250629.s009.docx]

Supplement table S 7 Mantel result comparison of parameters and diatom abundance

|  | Mantel test coefficient |
| --- | --- |
| Macroalgal host | 0.42*** |
| Macroalgal host class | 0.45 *** |
| Geographical effect | 0.30 *** |
| Depth effect | 0.26 *** |
| Antarctic Geographical effect | 0.46** |
